# Fatty Acid Profile, Total Phenolic Content, and Antioxidant Activity of Niger Seed (*Guizotia abyssinica*) and Linseed (*Linum usitatissimum*)

**DOI:** 10.3389/fnut.2021.674882

**Published:** 2021-08-02

**Authors:** Tesfaye Deme, Gulelat D. Haki, Nigussie Retta, Ashagrie Woldegiorgis, Mulatu Geleta

**Affiliations:** ^1^Department of Food Science and Applied Nutrition, Addis Ababa Science and Technology University, Addis Ababa, Ethiopia; ^2^Department of Food Science and Technology, Botswana University of Agriculture and Natural Resources, Gabarone, Botswana; ^3^Center for Food Science and Nutrition, College of Natural Sciences, Addis Ababa University, Addis Ababa, Ethiopia; ^4^Department of Plant Breeding, Swedish University of Agricultural Sciences, Alnarp, Sweden

**Keywords:** fatty acid, total phenolic content, varieties, niger seed, linseed, ferric reducing antioxidant power, DPPH, principal component analysis

## Abstract

Fatty acid composition and antioxidant content are major determinants of vegetable oil quality. Antioxidants are important food components, and there is an increasing interest of replacing synthetic antioxidants with those from natural sources for food industry. The objective of this study was to evaluate fatty acid composition, total phenolic, carotenoid and chlorophyll contents, and antioxidant capacity of different varieties of two oilseed crops. Five niger seed and eight linseed varieties were used. For the analysis of fatty acid composition of the seed oil, gas chromatography method was used. Standard methods were used for total phenolic, carotenoid and chlorophyll contents, and antioxidant properties. In niger seed oil, linoleic acid (C18:2) was the dominant fatty acid, accounting for 73.3% (variety *Esete*) to 76.8% (variety *Ginchi*) of the total fatty acids. In linseed oil, linolenic acid (C18:3) was the dominant fatty acid accounting for 55.7 (variety *Chilalo*) to 60.1 (variety *Belaye-96*). The total phenolic content ranged from 22.4 mg GAE/g (variety *Esete*) to 27.9 mg GAE/g (variety *Ginchi*) in niger seed and from 20.5 mg GAE/g (variety *Belay-96*) to 25.4 mg GAE/g (variety *Ci-1525*) in linseed. In niger seed, variety *Fogera* had the highest values for FRAP and radical scavenging activity. The carotenoid content also showed significant variation among the varieties ranging from 2.57 (*Esete*) to 8.08 (*Kuyu*) μmol/g for niger and 4.13 (*Tole*) to 8.66 (*Belay-96*) μmol/g for linseed. The FRAP assay showed that variety *Fogera* of niger seed and variety *Chilalo* of linseed came on top among their respective varieties with values of 57.2 and 30.6, respectively. Both niger seed and linseed were shown to be rich in bioactive compounds. However, significant variation was observed among the varieties of each crop and among the two crops in their total phenolic and carotenoid contents as well as ferric reducing potential and radical scavenging capacity. Principal component analysis revealed the presence of more than one group in both niger seed and linseed. Hence, genetic variation among the varieties should be utilized for improving their desirable characteristics through breeding. Both oil crops can be used as the source of antioxidants for replacing synthetic compounds.

## Introduction

Oilseed crops are vital sources of nutrients for both humans and animals in addition to their use for various industrial applications. The major edible oil crops that dominate the world market include soybean [*Glycine max* (L.) *Merr*.], rapeseed [*Brassica napus* (L.)], and sunflower [*Helianthus annuus* (L.)]. A rapid population growth has increased the demand for edible oilseed crops, which cannot be fulfilled based on a few major oilseed crops. Hence, increased production and utilization of other edible oilseed crops, in addition to the major ones, to satisfy the markets are highly important. Ethiopia is one of the major centers of origin and/or diversity of edible oilseed crops that include Ethiopian mustard (*Brassica carinata*), niger seed [*Guizotia abyssinica (L,f)* Cass.], sesame (*Sesamum indicum* L.) and linseed (*Linum usitatissimum* L.) (1). Various molecular marker based population genetics studies revealed high genetic diversity in these crops [e.g., (2–8)]. These crops are primarily used as sources of edible oil for local consumption. Niger seed, sesame and linseed covered 2.01% (ca 258 kha), 2.92% (ca 375 kha), and 0.54% (ca 69 kha) of the seed crop area and 0.87% (ca 2.92 kt), 0.78% (ca 2.63 kt), and 0.24% (ca 79 kt) of the grain production, respectively (9). Ethiopia exports different types of oilseeds to various countries, especially China, India and the European Union (10). Among these oil crops, sesame, niger seed, and linseed account for about 90, 6, and 1%, respectively. Besides increasing the production of these oilseed crops, there is a need to improve their nutritional qualities (11) so that it is possible to satisfy consumers' nutritional requirements.

Today's dietary trend is to gradually replace fats of animal origin with vegetable oil, including in countries where people predominantly consume animal fats. The change is related to the concept of a healthier lifestyle and the need to eat food with a positive influence on health; i.e., increasing the use of foods rich in proven beneficial components (12). Diets rich in saturated fatty acid (SFA) are a major risk factor for cardiovascular diseases (CVDs) because they lead to an increased serum low-density lipoprotein (LDL) cholesterol concentrations. On the other hand, monounsaturated fatty acids and some polyunsaturated fatty acids (PUFA) from vegetable oil, especially oleic acid (OA), linoleic acid (LA), and linolenic acid (LN) acid, play important roles in reducing or inhibiting CVDs (13–15). These fatty acids have a significant contribution to human health also through their chemopreventive properties against chronic or degenerative diseases (15, 16). The wound healing and skin barrier repairing ability of seed oils are attributed to synergistic effect of their antibacterial, antioxidant, and anti-inflammatory properties (17, 18). For example, due to its phenols, flavonoids and gamma tocopherol contents, roselle seed oil was considered as a potential new source of healthy edible oil (19). Similarly, Guo et al. (20) suggested that consumption of sunflower seed oil has a potential of reducing the risk of cardiovascular diseases and certain types of cancer, which is partly attributable to its tocopherol antioxidant activities.

Many plant species have been studied in search for new alternative sources of seed oils with preventive characteristics against numerous age-related degenerative diseases in human (15). Unlike many anti-inflammatory agents that have ulcerogenic effects, cold-pressed seed oils, such as those from coriander or black cumin are promising sources of anti-inflammatory agents that are highly desirable for treating inflammation (21). The anti-inflammatory activities of phenols, β-carotene, unsaturated fatty acids, and tocopherol in pumpkin seed oil have been demonstrated through their role in reducing the expression of inflammatory biomarkers in rat arthritis model (22).

Niger seed and linseed are among important edible oil crop rich in healthy long-chain polyunsaturated fatty acids and phenolic compounds. The oil content, fatty acid composition as well as other components of nutritional values of niger seed and linseed have been reported in a number of studies. The reports on niger seed fatty acid composition (23), variation and inheritance of fatty acid composition and oil content (24) and lipid classes and fatty acid profile (25, 26) are few examples. Similarly, the reports by Wakjira et al. (27) and Popa et al. (28) are examples of variation in fatty acid composition and oil content in linseed. Mono and polyunsaturated fatty acids account for about 90% of the fatty acids in both niger seed and linseed (29). Polyunsaturated fatty acids, such as linoleic acid are highly desirable in human diet because of their role in reducing the risk of coronary heart diseases and cancer (30). Oleic acid is known to reduce the levels of low-density lipoproteins and prevent atherosclerosis (30, 31). Vegetable oils that are high in the level of unsaturated fatty acids can easily undergo oxidation and produce unhealthy secondary products, especially when exposed to high temperature, such as during frying (32). However, the phenolic compounds in the seed oil contribute to its oxidative stability (31), and the health benefits of these compounds are related to their antioxidant activities (33). In line with this, for example, pomegranate (Lythraceae) oil was recognized as a natural source of bioactive compounds like phytosterols and tocopherols (34). Similarly the bioactive components like α-, β-, γ-tocopherols, carotenoids, campesterol, stigmasterol, β-sitosterol, catechin, caffeic acid in grape (Vitaceae) seed extract were shown to affect the thermal oxidation of soybean oil (35).

Pandey and Rizvi (36) reported that the presence of phenolic compounds in seed oils or their products is characterized by dark color, sour test, and undesirable flavors. The role of phenolic compounds as antioxidants include maintaining the quality and nutritional values of food by preventing or delaying its deterioration, and protecting body tissues from oxidative damage (37, 38). Their antioxidant mode of actions include preventing continued hydrogen abstraction from substrate and chelators of metal ions that are responsible for the formation of peroxidation compounds, reduction of oxygen concentration, terminating of free radicals, and decomposing primary products of oxidation to non-radical species (39). A study by Bhatnagar and Krishna (40) showed that niger seed of Indian origin has good antioxidant properties because of their bioactive components like phytosterols, carotenoids, tocopherols, and phenolics. Although these edible oil crops are known for their nutritional and health benefits, some highly important information is lacking, particularly for varieties grown in Ethiopia. Hence, the present study was conducted to evaluate the fatty acid profile, total phenolic content, and antioxidant properties of different varieties of niger seed and linseed grown in Ethiopia so that desirable varieties can be prioritized for production and further improvements.

## Materials and Methods

### Seed Sample Collection and Sample Preparation

In this study, five varieties of niger seed (*Esete, Fogera, Ginchi, Kuyu*, and *Shambu*) and eight varieties of linseed (*Bekoji, Belaye-96, Berene, Chilalo, Ci-1525, Ci-1652, Kassa-2*, and *Tole*) were used. The seed samples of all varieties of both crops were obtained from Holeta Agricultural Research Center, which is part of Ethiopian Institute of Agricultural Research (EIAR). All seeds used in this study were sampled from plants grown in the same location during the same year, to facilitate the comparison of the varieties of each crop as well as the two crops in terms of their contents. They were grown at Holeta experimental field station: soil type = vertisol, altitude = 2,400 m above sea level, temperature range = 6–22°C, and rainfall = 1,144 mm. According to the passport data obtained together with the seed samples, seed yield and oil content are among the major traits differentiating the different varieties of each crop.

All laboratory experiments were conducted at the Department of Plant Breeding, Swedish University of Agricultural Sciences (SLU), Alnarp, Sweden. Ten seeds per sample were used for fatty acid composition analyses in three replicates.

### Fatty Acid Composition

Total lipids of niger seed and linseed were extracted according to the method of Bligh and Dyer's (41) with some modifications as described in Geleta et al. (24). The ten seeds of each sample were weighed using sensitive balance before they were homogenized using the Ultra Turrax seed homogenizer for ~2 min in a mixture of 3.75 ml MeOH:CH_3_Cl_3_ (2:1, v/v) and 1 ml of 0.15 M acetic acid. To the homogenized sample, 1.25 ml chloroform and 0.9 ml water were added and thoroughly mixed by vortexing. The mixture was centrifuged for 2 min at 3,000 rpm to separate the mixture into clearly separated phases. The total volume of each extract is measured by transferring the bottom phase into new tubes. Then, 200 μl of each extract was transferred to a new glass tube and evaporated under a weak beam of nitrogen on a sand bath at 70°C temperature. For methylation of the fatty acids, 2 ml H_2_SO_4_ (2% in absolute methanol) was added to tubes containing the extracts and each mixture was incubated at 90°C for 45 min as described in Christie (42). Once the methylation of the fatty acid was completed and the temperature of the samples lowered down to room temperature, an internal standard of 100 ml of 1 nmol methyl-heptadecanoate (17:0-ME; Sigma–Aldrich Sweden AB) was added to the fatty acid methyl esters (FAMEs). This was followed by adding 1 ml Millipore water and 2 ml hexane (Merck KGaA; Germany) and thoroughly mixing by vortexing before the samples were centrifuged at 2,000 rpm for 2 min. For Gas Chromatography analysis, 200 μl of the hexane phase of each sample was transferred to the GC vials. To identify each FAME and to assess the accuracy of the analysis, a certified fatty acid methyl ester mixture *Me63* (Larodan Fine Chemicals AB; Sweden) was used as an external standard. The FAMEs were separated using a Shimadzu GC-17A V3 (Kyoto, Japan) equipped with a flame ionization detector (FID), an auto-sampler (AOC-20i + s) and a WCOT fused silica capillary column (50 m × 0.32 mm) coated with CP-wax 58 (FFAP)-CB (Varian Inc., Middelburg, the Netherlands) as described in Geleta et al. (24). The identification of FAMEs was based on the standards' retention times, and Shimadzu GC-solution Version 2.3 chromatography software (Kyoto, Japan) was used for peak integration by using the 17:0-ME peak area as a reference.

### Determination of Total Phenolic Content

The ultrasound-assisted extraction procedure described in Goli et al. (43) was used for sample extraction with slight modification. Briefly, 50 mg of crashed and lyophilized sample of each variety was extracted with 0.5 ml of ethanol in an ultrasonic bath for 45 min and centrifuged. Then, the supernatant was transferred to a new tube and the residue was re-extracted in the same way, and the supernatant was combined with the first extract and stored at −20°C until the analysis was conducted. The total phenolic content of each extract was determined according to the Folin-Ciocalteu method (44). Twenty microliter of each sample and the standard were mixed with 0.1 ml of Folin-Ciocalteu reagent and 1.58 ml of water, and then 0.3 ml of 7.5% saturated sodium carbonate was added. The mixture was incubated at room temperature in the dark for 2 h and the absorbance was measured at 765 nm, and the result was expressed as mg Gallic acid equivalents (GAE)/100 gm.

### Determination of Total-Carotenoid and Chlorophyll

The carotenoid and chlorophyll contents were determined according to the method described in Hendry and Grime (45) with some modifications. Briefly, 0.5 g of crashed and lyophilized oilseed sample was extracted with 10 ml of ethanol and hexane mixture (4:3 v/v) in an ultrasonic bath for 60 min. Butylated hydroxytoluene (BHT) was added to the extract to prevent oxidation before it was centrifuged and the supernatant was collected. The absorbance of the extract was recorded using Thermo scientific (Multiskan Go) spectrophotometer at 480, 645, 663, and 710 nm. The total carotenoid, chlorophyll a, chlorophyll b and total chlorophyll were estimated using the equation 1, 2, 3 and 4 shown below in that order.

$$\begin{array}{r} Carotenoid\left(\frac{\mu mol}{g}\right)= \end{array}$$

(1) $$\begin{array}{r} \frac{\left(A480+0.114\;x\;A663-0.638\;x\;A645\right)x\;V\;x\;1000}{112.5\;x\;weight} \end{array}$$

where *V* = volume of extract.

(2) $$\begin{array}{r} Chlorophyll\;a\;\left(\frac{mg}{l}\right)=12.7\;x\;A663-2.69\;x\;A645 \end{array}$$

(3) $$\begin{array}{r} Chlorophyll\;b\left(\frac{mg}{l}\right)=22.9\;x\;A645-4.68\;x\;A663 \end{array}$$

(4) $$\begin{array}{r} Total\;chlorophyll\;\left(\frac{mg}{l}\right)=8.02\;x\;A663+20.2\;x\;A645 \end{array}$$

### Determination of Ferric Reducing Antioxidant Power

Ferric reducing antioxidant power (FRAP) was determined using the method described in Benzie and Strain (46) and Perez-Jimenez and Saura-Calixto (47) with slight modification. Briefly, 50 mg of freeze-dried samples were extracted in 1 ml of 50% methanol, pH 2, for 1 h on a shaker at 1,000 rpm, which was then centrifuged at 8,000 rpm for 10 min. The supernatant was transferred to a new e-tube and the pellet was re-extracted with 1 ml acetone: water (70:30 v/v) in the same way. The supernatants were combined and stored at −20°C until use for the analysis. For the assay, 300 mM acetate buffer (pH 3.6), 10 mM TPTZ (2,4,6-tripyridyl-s-triazine) in 40 mM HCl and 20 mM FeCl_3_ in distilled water were prepared and used in the proportion of 10:1:1. Two-hundred microliter of this freshly prepared reagent (kept at 37°C) was added to 20 μl of each sample and the standard solutions (0–2 mM). Then, the absorbance of the samples and the standard was measured two times at 593 nm, and quantification was made based on the standard solutions.

### Radical Scavenging Activity by DPPH (2,2-diphenyl-1-picrylhydrazyl) Assay

The protocol of Perez-Jimenez and Saura-Calixto (47) were used for 2,2-diphenyl-1-picrylhydrazyl (DPPH) assay, with slight modifications. Briefly, 50 mg of each sample was extracted in 1 ml methanol for 3 h on a shaker at 150 rpm, and then centrifuged at 3,000 rpm for 10 min. The supernatant was transferred to a new test tube and the pellet was re-extracted in the same way. The supernatants were combined and stored at −20°C till use for the assay.

The DPPH assay measures hydrogen atom (or an electron) donating activity and provides an estimate of free-radical scavenging antioxidant activity. During this process, the purple-colored stable free radical DPPH is reduced to the yellow-colored diphenylpicrylhydrazine (48). The effect of methanolic extracts on DPPH radical was estimated according to Kirby and Schmidt (48). For the assay, 4 ml of 0.004% solution of DPPH in methanol was mixed with 1 ml of each extract at various concentrations (1–10 mg/ml methanol). Then, the samples were incubated for 30 min in the dark at room temperature. The scavenging capacity was read spectrophotometrically (Perkin Elmer Lamda 950 UV/Vis/NIR) by monitoring a decrease in absorbance at 517 nm. Ascorbic acid was used as a standard, and a mixture without the extracts was used as a control. The percentage inhibition of DPPH (I%) was then calculated as:

(5) $$\begin{array}{r} Radical\;scavenging\;activity\;\left(\%I\right)=\frac{A0-A1}{A0}\;x\;100 \end{array}$$

Where A_0_ is the absorbance of the control and A_1_ is the absorbance of the sample. The extract concentration providing 50% of the radical's scavenging activity (EC_50_) was calculated from the graph of percent radical scavenging activity (RSA) against the concentration of the extracts (49).

### Statistical Analysis

Descriptive statistical analyses were done and means of triplicate measurements were presented for the fatty acid profiles. One-way analysis of variance (ANOVA) followed by Duncan's Test at 95% level of significance were done for total antioxidant contents and properties; and the results were expressed as mean values and their standard deviation (SD) of triplicate measurements. SPSS version 20.0 package (SPSS, IBM Corp., Chicago, IL, USA) was used for ANOVA whereas XLSTAT software version 2015.5.01.22537 was used for principal component analysis (PCA).

## Results and Discussion

### Fatty Acid Composition

The major fatty acids in niger seed are linoleic, oleic, palmitic, and stearic acids. Linoleic acid (C18:2) was the principal unsaturated fatty acid in all niger seed varieties included in the present study ranging from 73.3 to 76.8% (Table 1). Oleic acid (C18:1) was the second major unsaturated fatty acid accounting for 4.7 to 6.1% of the total fatty acids. Palmitic acid (C16:0) and stearic acid (C18:0) were the major saturated fatty acids accounting for 7.6 to 8.3% and from 6.1 to 7.1% of the total fatty acids, respectively. These four fatty acids (linoleic, oleic, palmitic, and stearic) constituted about 96% of the total fatty acids in niger seed oil. In addition to these major fatty acids, minor fatty acids that include myristic (C14:0), palmitoleic (C16:1), linolenic (C18:3), arachidic (C20:0), Eicosenoic (C20:1n-11 and C20:1n-13), behenic (22:0), erucic (C22:1), and lignoceric (C24:0) were identified in niger seed oil, and together constituted about 1.8% of the total fatty acids. This result is in agreement with that of Geleta et al. (24) for the major fatty acids. The level of behenic acid (C22:0) found in the present study is similar to that reported by Ramadan and Mörsel (25). The fatty acids were grouped into saturated, monounsaturated, and polyunsaturated fatty acids for further characterization and comparison of the oils of the two crops. In niger seed, the contribution of saturated fatty acids (SFA) ranged from 15.3% (variety *Ginchi*) to 17.2% (variety *Esete*). Similarly, the monounsaturated fatty acids (MUFA) accounted for 4.7% (variety *Ginchi*) to 7.3% (variety *Esete*), which needs be exploited to increase the content of MUFA as they enhance the oxidative stability of the oil (50). Increased MUFA, such as C18:1 in edible oil are desirable due to their hypocholesterolemic effect (51).Unlike the MUFA, the polyunsaturated fatty acids (PUFA) ranged from 73.5 (variety *Esete*) to 77.0 (variety *Ginchi*). The major PUFA in niger seed oil is C18:2, which is an omega-6 essential fatty acid that cannot be synthesized in the human body, and hence must be obtained through diet for human health (52).

**Table 1:** Fatty acid composition (%) of the different niger seed varieties.

**Fatty acid**	**Niger seed varieties**				
	**Shambu**	**Kuyu**	**Esete**	**Ginchi**	**Fogera**
C14:0	0.05	0.05	0.05	0.04	0.04
C16:0	8.06	8.02	8.29	7.58	8.29
C16:1	0.09	0.11	0.11	0.10	0.09
C18:0	7.13	6.58	7.02	6.13	6.35
C18:1	6.06	6.59	6.25	4.63	4.68
C18:2	76.43	75.43	73.32	76.84	75.07
C18:3	0.22	0.21	0.17	0.19	0.19
C20:0	0.4	0.41	0.49	0.41	0.41
C20:1 (11c)	0.04	0.06	0.04	0.05	0.05
C20:1 (13c)	0.04	0.06	0.05	0.07	0.05
C22:0	0.55	0.57	0.89	0.72	0.95
C22:1 (13c)	0.04	0.13	0.83	0.37	0.76
C24:0	0.38	0.45	0.48	0.43	0.38
SFA	16.57	16.08	17.22	15.31	16.42
MUFA	6.27	6.95	7.28	4.72	5.63
PUFA	76.65	75.64	73.49	77.03	75.26

*SFA, Saturated fatty acids; MUFA, Monounsaturated fatty acids; PUFA, Polyunsaturated fatty acids*.

The fatty acid profile for different varieties of linseed is presented in Table 2. The principal fatty acids in linseed are linolenic acid (C18:3), oleic acid (C18:1), linoleic acid (C18:2), palmitic acid (C16:0), and stearic acid (C18:0) with percent composition ranging from 55.7 to 60.1, 15.9 to 20.3, 13.3 to 16.6, 4.9 to 8.1, and 3.3 to 4.5% of the total fatty acids, respectively. On average, linolenic, oleic, linoleic, palmitic and stearic acids accounted for 57.2%, 17.7%, 14.5% and 5.6%, respectively. These results are similar to those reported by Choo et al. (53), Popa et al. (28), and Teh and Birch (54). The levels of minor fatty acids were also in agreement with the reports of Choo et al. (53), and Teh and Birch (54). At a group-level, the percentage of SFA ranged from 8.7% (variety *Kassa-2*) to 12.7% (variety *Bokoji*). Likewise, the MUFA accounted for 16.1% (variety *Belaye-96*) to 20.5% (variety *Berene*) of the total fatty acids. Similar to the case in niger seed, the PUFA are dominant in linseed oil accounting for 69.0% (variety *Chilalo*) to 73.7% (variety *Belaye-96*) although the dominant fatty acid is alpha-linolenic acid, which is an omega-3 essential fatty acid. Although oil rich in PUFA has several health benefits, it has a relatively low oxidative stability and thermostability, and hence its applications in some areas are limited, e.g., for use as frying oil. Since both niger seed and linseed are major edible oil crops in countries likes Ethiopia, developing varieties with elevated levels of MUFA (e.g., oleic acid) through breeding is desirable for better shelf-life and thermostability of the oil (24).

**Table 2:** Fatty acid composition (%) of the linseed varieties.

**Fatty acid**	**Linseed varieties**							
	**Chilalo**	**Tole**	**Ci-1652**	**Kassa-2**	**Bekoji**	**Berene**	**Ci-1525**	**Belaye-96**
16:0	5.24	5.05	5.34	4.92	8.12	5.41	5.33	5.16
18:0	4.39	3.79	3.94	3.33	4.02	3.83	4.54	4.47
18:1	17.26	17.68	16.38	17.63	17.06	20.33	19.29	15.93
18:2	13.31	14.69	14.28	16.62	13.83	15.02	14.34	13.68
18:3	55.67	57.2	56.49	56.63	58.96	56.67	56.23	60.05
20:0	0.15	0.14	0.15	0.13	0.11	0.09	0.08	0.08
20:1 (11c)	0.1	0.11	0.1	0.11	0.28	0.13	0.22	0.15
22:0	0.14	0.12	0.13	0.11	0.26	0.16	0.19	0.22
24:0	0.40	0.45	0.40	0.37	0.20	0.12	0.22	0.23
SFA	10.32	9.52	9.96	8.86	12.71	9.61	10.36	10.16
MUFA	17.36	17.79	16.48	17.74	17.34	20.46	19.51	16.08
PUFA	68.98	71.89	70.77	73.25	72.79	71.69	70.57	73.73

*SFA, Saturated fatty acids; MUFA, Monounsaturated fatty acids; PUFA, Polyunsaturated fatty acids*.

### Total Phenolic, Carotenoid, and Chlorophyll Contents and Antioxidant Properties (FRAP and DPPH)

The total antioxidant contents and properties of niger seed are presented in Table 3. The total phenol, carotenoid, and total chlorophyll contents were in the range of 22.41–27.88 mg GAE/g, 2.57–8.08 μmol/g, and 0.41–1.68 mg/l, respectively. Similarly, the values for FRAP, chlorophyll a, and b of niger seed were in the range of 42.86–57.22 mmol Fe_2_SO_4_/100 g, 0.084–0.23 mg/l, and 0.24–1.44 mg/l. The total phenolic content was the highest in variety *Ginchi* (27.8.8 mg GAE/100 g) and the lowest in variety *Esete* (224.1mg GAE/100 g), suggesting significant differences between the varieties in this trait. A similar finding was reported for olive mill wastewaters (55, 56). According to Allouche et al. (56), the occurrence of specific bio-phenols in olive mill wastewater varied with the fruit type (e.g., cultivar and maturity), climatic conditions, and storage time, in addition to the processing techniques. Mulinacci et al. (55) examined varietal and processing effects using olive mill wastewater collected from commercial and experimental mills with different varieties from four European countries, and showed that total antioxidant contents and properties varied between the varieties. The finding of this study is also in line with the report of Bhatnagar and Krishna (57) for commercial Indian niger seed with a slight difference in some of the varieties. However, the values obtained in this study are higher than those reported for niger seed grown in India by Dande and Manchala (58). Studies have shown that the antioxidant contents and properties vary for various reasons, such as growing conditions, seasonal changes, genetic variation between cultivars, storage conditions, and differences in the extraction procedures (59).

**Table 3:** Total antioxidant contents and properties of the niger seed varieties.

**Parameters**	**Niger seed varieties**				
	**Shambu**	**Kuyu**	**Esete**	**Ginchi**	**Fogera**
Total phenol (mg GAE/g)	27.47^a^ ± 1.6	24.33^ab^ ± 2.4	22.41^b^ ± 0.2	27.88^a^ ± 1.49	27.54^a^ ± 0.86
Carotenoid (μmol/g)	6.47^b^ ± 0.1	8.08^a^ ± 0.82	2.57^d^ ± 0.35	5.54^bc^ ± 0.62	4.58^c^ ± 0.20
FRAP (mmol Fe_2_SO_4_/100g)	53.1^ab^ ± 0.1	52.72^b^ ± 1.2	47.83^c^ ± 0.3	42.86^d^ ± 2.2	57.22^a^ ± 1.4
Chlorophyll A / (mg/l)	0.084^a^ ± 0.0	0.137^a^ ± 0.01	0.23^a^ ± 0.02	0.15^a^ ± 0.01	0.13^a^ ± 0.004
Chlorophyll B (mg/l)	0.24^c^ ± 0.00	1.44^a^ ± 0.06	1.32^a^ ± 0.07	1.36^a^ ± 0.12	1.09^b^ ± 0.05
Total chlorophyll (mg/l)	0.41^c^ ± 0.01	1.56^a^ ± 0.07	1.38^b^ ± 0.00	1.25^b^ ± 0.06	1.68^a^ ± 0.07

*Means followed by different superscripts in the same row are significantly different (P < 0.05)*.

The total phenolic content of the linseed varieties ranged from 20.53 mg GAE/g in variety *Belaye-96* to 25.41 mg GAE/g in variety *Ci-1525*. Similarly, significant variation was observed for the total carotenoid content among the linseed varieties with values ranging from 4.13 μmol/g in variety *Tole* to 8.66 μmol/g in variety *Belay-96* (Table 4). The ferric reducing antioxidant potential (FRAP) values of the methanolic extracts of linseed was within the range of 15.28 (for variety *Kass-2*) to 30.59 (for variety *Chilalo*) mmol Fe_2_SO_4_/100 g. The total chlorophyll content of the linseed varieties showed over two-fold variation with the values ranging from 1 mg/l in *Belaye-96* to 2.36 mg/l in *Berene*. However, these values are lower than those reported by Tuberoso et al. (12). Chlorophyll content of seed oils is usually associated with oil quality, for example in olive oil. However, its high concentration can negatively affect the oil stability (60). Both carotenoid and chlorophyll play a role as antioxidants; however, they also act as pro-oxidants because of their instability against light and heat (61) and hence their high concentrations should be avoided. The variation in the antioxidant levels among the different varieties of both crops provides opportunity to improve the oil quality through further breeding.

**Table 4:** Total antioxidant contents and properties of the linseed varieties.

**Parameters**	**Linseed varieties**							
	**Chilalo**	**Tole**	**Ci-1652**	**Kassa-2**	**Bekoji**	**Berene**	**Ci-1525**	**Belaye-96**
Total phenol (mg GAE/g)	22.52^ab^ ± 0.11	22.77^ab^ ± 0.05	24.22^a^ ± 0.17	23.49^ab^ ± 0.12	24.74^a^ ± 0.09	20.79^b^ ± 0.17	25.41^a^ ± 0.05	20.53^b^ ± 0.15
Total carotenoid (μmol/g)	7.34^b^ ± 0.26	4.13^d^ ± 0.05	4.42^d^ ± 0.45	7.46^b^ ± 0.73	7.26^b^ ± 0.37	6.25^c^ ± 0.11	5.45^c^ ± 0.68	8.66^a^ ± 0.15
FRAP (mmol Fe_2_SO_4_/100g)	30.59^a^ ± 0.23	30.17^a^ ± 0.81	27.12^b^ ± 0.13	15.28^e^ ± 0.08	17.38^d^ ± 0.89	19.95^c^ ± 1.48	17.30^d^ ± 0.15	18.59^cd^ ± 1.22
Chlorophyll A (mg/l)	0.18^bc^ ± 0.01	0.08^e^ ± 0.01	0.10^e^ ± 0.01	0.14^d^ ± 0.02	0.15^cd^ ± 0.01	0.20^ab^ ± 0.02	0.10^e^ ± 0.01	0.23^a^ ± 0.01
Chlorophyll B (mg/l)	1.56^d^ ± 0.05	1.62^d^ ± 0.00	1.72^c^ ± 0.01	1.79^c^ ± 0.01	1.93^b^ ± 0.01	1.99^b^ ± 0.07	1.80^c^ ± 0.03	2.08^a^ ± 0.06
Total chlorophyll (mg/l)	1.64^f^ ± 0.01	1.70^ef^ ± 0.05	1.82^e^ ± 0.01	1.93^d^ ± 0.05	2.08^c^ ± 0.06	2.19^b^ ± 0.02	1.90^d^ ± 0.05	2.31^a^ ± 0.01

*Means followed by different superscripts in the same row are significantly different (P < 0.05)*.

### The Free Radical Scavenging Activity

**Figure 1:**
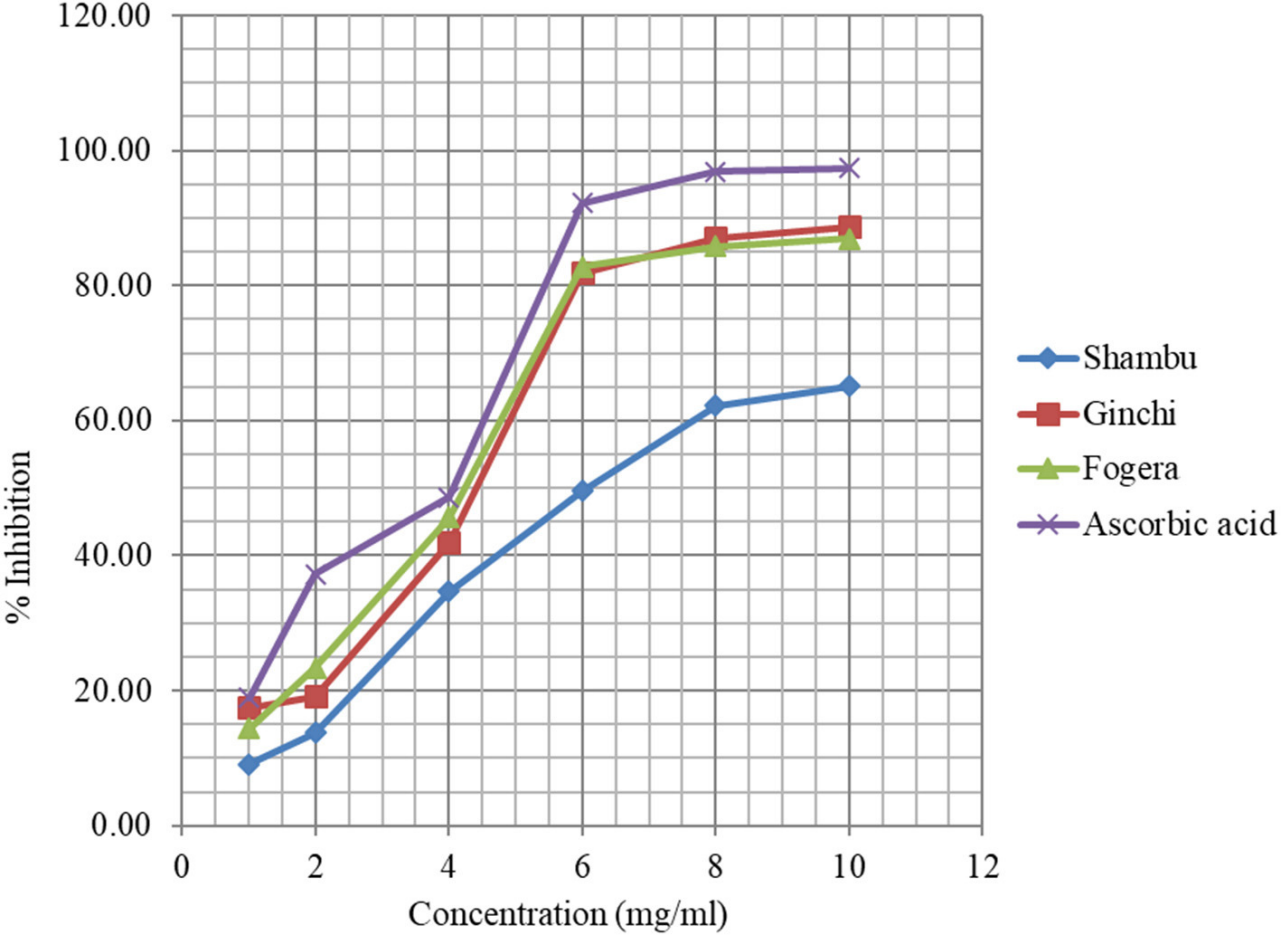
Free radical scavenging of methanolic extracts of three niger seed varieties and the control.

The DPPH assay based free radical scavenging capacity of methanol extracts of three of the five niger seed varieties with higher total phenolic content is presented in Figure 1. The percent inhibition was shown to be dependent on the concentration of the extracts, and vary among the varieties, with variety *Shambu* exhibiting the lowest inhibition capacity. The percent inhibition capacity of variety *Ginchi* and *Fogera* at 4 mg/ml concentration was comparable with that of the control. Although the three varieties had similar total phenolic content, variety *Shambu* scored the lowest percent inhibition. Bhatnagar and Krishna (57) studied the effect of extraction solvent on oil and bioactive composition of commercial Indian niger seed. The RSA of niger seed extracted with acetone, methanol, and ethanol could be attributed to the presence of high amounts of bioactive molecules such as tocopherols, phenolics, sterols, and carotenoids. Apart from these, the presence of Milliard reaction products extracted by the polar solvents could also be contributing to the increased radical scavenging activity. The EC_50_ (sample concentrations causing 50% reduction in a total amount of DPPH radicals) of variety *Ginchi* and *Fogera* was comparable with the reference, suggesting the possibility of replacing synthetic antioxidants with those extracted from oilseeds.

**Figure 2:**
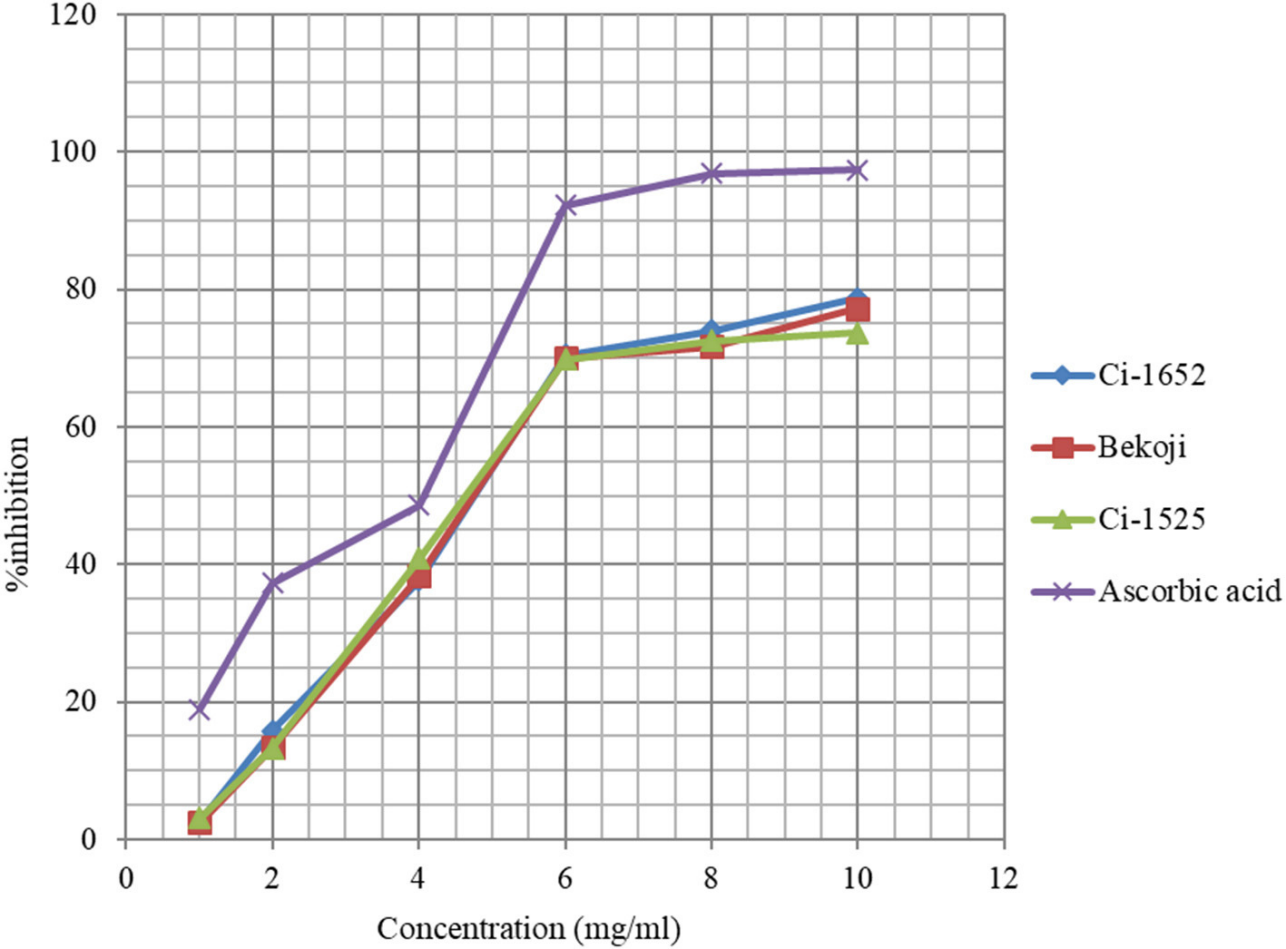
Free Radical scavenging of methanolic extracts of three linseed varieties and the control.

The DPPH assay based free radical scavenging capacity of methanol extracts of three of the eight linseed varieties with higher total phenolic content is presented in Figure 2. The ability of linseed extracts to scavenge the DPPH radical was increased from 2.7 to 78% when the concentration of the extracts was increased from 1 to 10 mg/ml, hence the higher the concentration of extracts the higher the capacity to scavenge the free radicals. Interestingly, the three varieties showed similar scavenging capacity at each concentration level unlike the case for niger seed. The EC_50_ (4.75 mg/ml) of linseed was higher than that of niger seed, and the radical scavenging capacity of the niger seed extracts were relatively higher than that of linseed, which can be partly explained by slightly higher total phenolic content in niger seed (Tables 3, 4). The polar lipids like glycolipids and phospholipids in niger seed oil (62) may have also contributed to its higher antioxidant property.

Amin and Thakur (63) reported that 10 mg/ml linseed extract had 32.33% inhibition while the inhibition was 82.53% at 50 mg/ml. In the present study, the lowest concentration we used was 1 mg/ml corresponding to 2.7% inhibition and the highest was 10 mg/ml corresponding to 78% inhibition. They reported the EC_50_ value of 25.63 mg/ml for ethanolic extract of linseeds, which is about six-fold higher than the result obtained in this study for linseed (ca 4.75 mg/ml). Ramadan et al. (64) indicated that different factors may contribute to the difference in the strength of RSA. These include differences in content and composition of polar lipids and unsaponifiables, diversity in structural characteristics of phenolic antioxidants and synergism of polar lipids with other components.

### Principal Component Analysis

**Figure 3:**
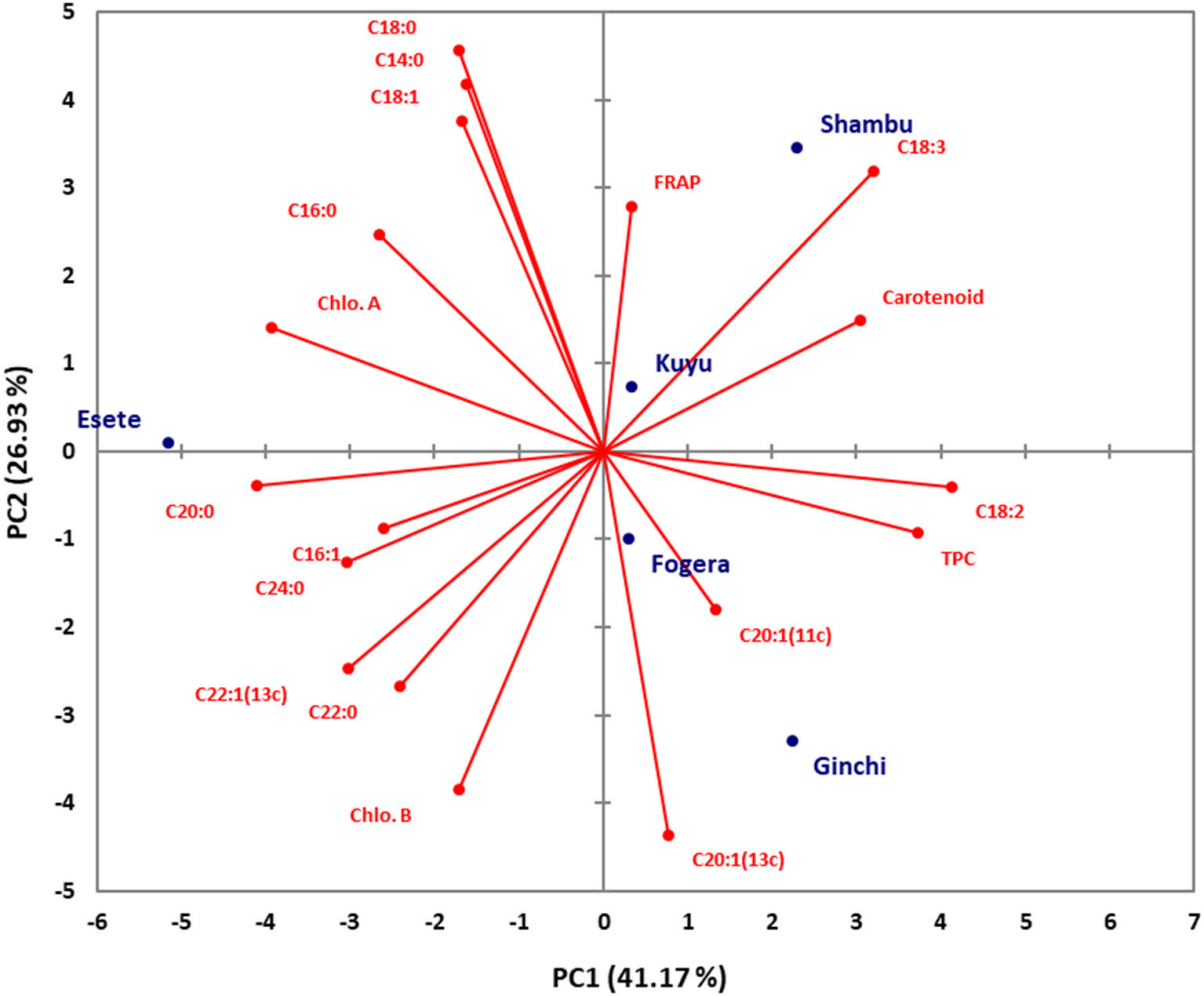
Score plot of principal component analysis (PCA) for the combined data sets of fatty acid composition, total phenolic, carotenoid, and chlorophyll contents and their antioxidant properties for the niger seed varieties.

**Figure 4:**
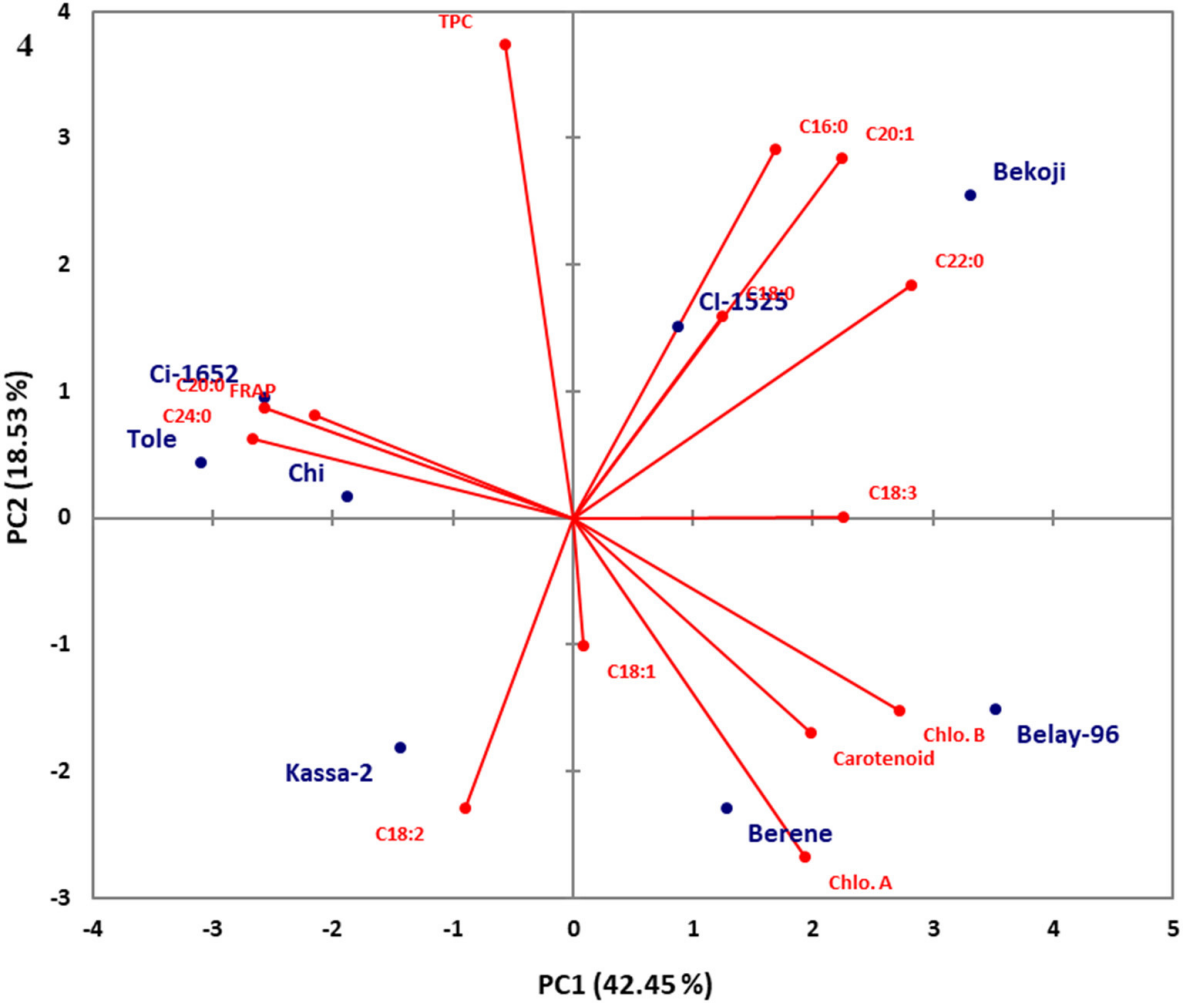
Score plot of principal component analysis (PCA) for the combined data sets of fatty acid composition, total phenolic, carotenoid, and chlorophyll contents and their antioxidant properties for the linseed varieties.

Principal component analysis (PCA) was conducted using combined data sets of fatty acid composition, total phenolic, carotenoid and chlorophyll contents as well as their antioxidant properties for both niger seed and linseed varieties (Figures 3, 4). In the case of niger seed, the first and second principal components (PCs) together explained 68.1% of the total variation with PC1 and PC2 explaining 41.2 and 26.9%, respectively. Similarly, PC1 and PC2 explained 42.4% and 18.5% in linseed, and hence the two PCs explained 61% of the total variation. In niger seed, two distinct groups were formed, mainly along the PC1 axis, with variety *Esete* separated from the other four varieties. Fatty acid and chlorophyll profiles are the major contributors to the separation of variety *Esete*, with arachidic acid (C20:0) and chlorophyll a contributing the most. Variety *Shambu* and *Ginchi* were separated along the PC2 axis (within the second group) mainly due to their differences in linolenic acid (C18:3) and eicosenoic acid (C20:1) contents. Similarly, the eight linseed varieties formed two major groups: group-1 (*Ci-1652, Tole, Chilalo*, and *Kassa-2*) and group-2 (*Bekoji, Ci-1525, Belaye-96*, and *Berene*) along the PC1 axis that contributed 42.4% of the total variation. Within group-1, linoleic acid (C18:2) was the major contributor to the separation of variety *Kassa-2* from the other three varieties, which had similar profile in FRAP, arachidic acid (C20:0), and lignoceric acid (C24:0). Group-2 formed two sub-groups along the PC2 axis with variety *Bekoji* and *Ci-1525* forming the first sub-group and variety *Belay-96* and *Berene* forming the second sub-group. Chlorophyll and carotenoid contents are the major contributors to the separation of the second sub-group.

The first four PCs explained 100% of the variance among the five varieties of niger seed with PC1–PC4 having eigenvalues of 7.41, 4.85, 3.93, and 1.81 in that order (Table 5). These numbers denote important contributions of each PC to the total variance. PC1 explained 41.17% of the variance in the data set, and its loadings indicated that it has high contributions from palmitic acid (C16:0; −0.624), palmitoleic acid (C16:1; −0.609), linoleic acid (C18:2; 0.968), linolenic acid (C18:3; 0.752), arachidic acid (C20:0; −0.963), erucic acid (C22:1; −0.708), lignoceric acid (C24:0; −0.712), TPC (0.877), carotenoid (0.717), and chlorophyll a (−0.922). Similarly, PC2 showed top positive loadings for myristic acid (C14:0; 0.793), stearic acid (C18:0; 0.866), oleic acid (C18:1; 0.714), and linolenic acid (C18:3; 0.606) whereas top negative loadings came from eicosenoic acid [C20:1(13c); −0.828] and chlorophyll b (−0.731). The top contributors to PC3 were palmitoleic acid (C16:1; 0.775), arachidic acid (C20:0; −0.631) and lignoceric acid (C24:0; 0.644) whereas eicosenoic acid [C20:1(11c); 0.711] and FRAP (0.729) were top contributors to PC4.

**Table 5:** Eigen analysis of the loadings of the significant principal components (PCs) for the niger seed varieties.

**Parameters**	**PC1**	**PC2**	**PC3**	**PC4**
C14:0	−0.381	0.793	0.464	−0.102
C16:0	−0.624	0.469	−0.454	0.430
C16:1	−0.609	−0.166	0.775	0.034
C18:0	−0.401	0.866	−0.026	−0.299
C18:1	−0.394	0.714	0.576	0.054
C18:2	0.968	−0.076	0.110	−0.211
C18:3	0.752	0.606	0.218	0.142
C20:0	−0.963	−0.073	0.020	−0.258
C20:1 (11c)	0.314	−0.342	0.528	0.711
C20:1 (13c)	0.181	−0.828	0.531	0.012
C22:0	−0.564	−0.508	−0.631	0.163
C22:1 (13c)	−0.708	−0.469	−0.518	0.104
C24:0	−0.712	−0.239	0.644	−0.145
TPC	0.877	−0.175	−0.440	−0.080
Carotenoid	0.717	0.282	0.547	0.327
FRAP	0.080	0.529	−0.427	0.729
Chlorophyll a	−0.922	0.269	−0.016	0.279
Chlorophyll b	−0.402	−0.731	0.450	0.320
Eigenvalue	7.41	4.85	3.93	1.81
Variability (%)	41.17	26.93	21.85	10.05
Cumulative (%)	41.17	68.1	89.95	100.00

Unlike niger seed in which the compositional data is distributed under four components, the total variance was distributed in seven PCs in the case of linseed. The first four PCs explained 89.32% of the variance in the data set (Table 6), and PC5 to PC7 explained the remaining 10.68% of the total variance. However, according to Kaiser's rule, only those with eigenvalues >1 were considered. The eigenvalues for the first four PCs were 5.94, 2.59, 2.43, and 1.53 in that order. PC1 showed top positive loadings for linolenic acid (C18:2; 0.729), eicosenoic acid (C20:1; 0.723), behenic acid (C22:0; 0.911), carotenoid (0.639), chlorophyll a (0.625), and chlorophyll b (0.879) and top negative loadings for arachidic acid (C20:0; −0.829), lignoceric acid (C24:0; −0.861) and FRAP (−0.696). PC2 explained 18.53% of the variance in the data set, and its loadings indicated that it has high contributions from palmitic acid (C16:0; 0.621), eicosenoic acid (C20:1; 0.607) and TPC (0.798). Oleic acid (C18:1) was a top contributor to both PC3 (0.690) and PC4 (0.618). Stearic acid (C18:0) was also a top contributor to PC4 (0.605).

**Table 6:** Eigen analysis of the loadings of the significant principal components (PCs) for the linseed varieties.

**Parameters**	**PC1**	**PC2**	**PC3**	**PC4**
C16:0	0.544	0.621	0.094	−0.301
C18:0	0.402	0.34	−0.499	0.605
C18:1	0.029	−0.215	0.69	0.618
C18:2	−0.288	−0.49	0.723	−0.374
C18:3	0.729	0.002	−0.314	−0.433
C20:0	−0.829	0.185	−0.263	−0.335
C20:1	0.723	0.607	0.294	−0.031
C22:0	0.911	0.394	−0.109	−0.014
C24:0	−0.861	0.133	−0.317	−0.301
TPC	−0.183	0.798	0.447	−0.118
Carotenoid	0.639	−0.362	−0.311	−0.279
FRAP	−0.696	0.174	−0.564	0.247
Chlorophyll a	0.625	−0.572	−0.42	0.045
Chlorophyll b	0.879	−0.324	0.129	−0.102
Eigenvalue	5.94	2.59	2.43	1.53
Variability (%)	42.45	18.53	17.37	10.96
Cumulative (%)	42.45	60.98	78.35	89.32

The principal component loadings (Tables 5, 6) demonstrated the extent to which the analyzed variables influenced the grouping of the varieties as well as the degree of association among the variables. In both niger seed and linseed, the loading projections visualized the position of the variables and the varieties in the two-dimensional plot and their corresponding correlations. Variables closest to one another and far from the plot origin are positively correlated. Hence, for example, the results suggest positive correlation between chlorophyll a and palmitic acid (C16:0) as well as between chlorophyll b and behenic acid (C22:0) in niger seed (Figure 3). In linseed, Chloropyll a and b contents appeared to be positively correlated with carotenoid content (Figure 4).

## Conclusion

The two oilseed crops showed major differences in several characteristics although both are rich in bioactive compounds. The principal fatty acids in niger seed oil is linoleic acid (C18:2), which accounted for over 73% of the total fatty acids whereas linolenic acid (C18:3) is the principal fatty acid in linseed accounting for over 55% of the total fatty acids. Significant variation exist within the varieties of each crop as well as among them in their total phenolic content, carotenoid content, ferric reducing potential, and DPPH scavenging capacity. Taking total phenolic content, FRAP and radical scavenging activity into account, variety *Fogera* can be selected among niger varieties for its antioxidant properties for further improvement. Similarly, *Ci-1525* is a preferred linseed variety in terms of total phenolic content. The antioxidant capacity of the seed extracts of the two crops, particularly those from niger seed varieties, suggest their potential to replace synthetic compounds. The varieties of both crops formed more than one group as revealed by principal component analysis suggesting the presence of genetic variation for improving desirable traits considered in the present study.

## Data Availability Statement

The original contributions generated for the study are included in the article/supplementary material, further inquiries can be directed to the corresponding author/s.

## Author Contributions

TD and MG designed and conducted the experiments, and analyzed the data. TD wrote the manuscript. MG, GH, NR, and AW revised the manuscript. All coauthors participated in conceiving the experiments and interpreting the data.

## Conflict of Interest

The authors declare that the research was conducted in the absence of any commercial or financial relationships that could be construed as a potential conflict of interest.

## Publisher's Note

All claims expressed in this article are solely those of the authors and do not necessarily represent those of their affiliated organizations, or those of the publisher, the editors and the reviewers. Any product that may be evaluated in this article, or claim that may be made by its manufacturer, is not guaranteed or endorsed by the publisher.
